# The Role of Gut Microbiota in Gestational Diabetes Mellitus Affecting Intergenerational Glucose Metabolism: Possible Mechanisms and Interventions

**DOI:** 10.3390/nu15214551

**Published:** 2023-10-27

**Authors:** Yaolin Ren, Yuan Zeng, Yifan Wu, Jie Yu, Qian Zhang, Xinhua Xiao

**Affiliations:** 1 Key Laboratory of Endocrinology, Ministry of Health, Department of Endocrinology, Peking Union Medical College Hospital, Peking Union Medical College, Chinese Academy of Medical Sciences, Beijing 100730, China; wuhanryl@163.com (Y.R.); drzeng99@163.com (Y.Z.); wuyifan5151@163.com (Y.W.); yujiedoctor@163.com (J.Y.); 2 State Key Laboratory of Complex Severe and Rare Diseases, The Translational Medicine Center of Peking Union Medical College Hospital, Chinese Academy of Medical Sciences, Beijing 100730, China

**Keywords:** diabetes mellitus, gestational diabetes mellitus, gut microbiota, Developmental Origins of Health and Disease, intergenerational effect

## Abstract

The incidence of type 2 diabetes is increasing every year and has become a serious public health problem. In addition to genetic factors, environmental factors in early life development are risk factors for diabetes. There is growing evidence that the gut microbiota plays an important role in glucose metabolism, and the gut microbiota of pregnant women with gestational diabetes mellitus (GDM) differs significantly from that of healthy pregnant women. This article reviews the role of maternal gut microbiota in offspring glucose metabolism. To explore the potential mechanisms by which the gut microbiota affects glucose metabolism in offspring, we summarize clinical studies and experimental animal models that support the hypothesis that the gut microbiota affects glucose metabolism in offspring from dams with GDM and discuss interventions that could improve glucose metabolism in offspring. Given that adverse pregnancy outcomes severely impact the quality of survival, reversing the deleterious effects of abnormal glucose metabolism in offspring through early intervention is important for both mothers and their offspring.

## 1. Introduction

In recent years, the incidence of diabetes has been on the rise. In 2021, 10.5% of adults worldwide had diabetes; it is predicted that by 2024, 783.2 million people worldwide will be affected by diabetes [1]. The etiology and pathogenesis of diabetes are complex, involving both environmental and genetic factors; Barker’s early “thrifty phenotype” hypothesis suggests that there is a correlation between the early developmental environment and the prevalence of metabolic disease in offspring and that the risk of metabolic disease in offspring increases in the presence of unfavorable environmental factors in early development, which is the basis of the Developmental Origins of Health and Disease (DOHaD) theory [2]. Numerous early-life environmental factors affect metabolic health in adults, including maternal obesity before pregnancy, gut microbiota disorders, gestational diabetes mellitus (GDM), malnutrition, and short sleep duration in early childhood. Both gestational diabetes and the gut microbiota have a significant impact on the health of offspring later in life.

In 1952, Professor Jorgen Pedersen hypothesized that maternal hyperglycemia would lead to fetal hyperglycemia, thereby stimulating insulin overproduction in the fetus to maintain normal glycemia. The “Jorgen Pedersen hypothesis” provides a pathophysiological basis for understanding the adverse pregnancy outcomes of hyperglycemia during pregnancy; however, the pathogenesis of the profound effects of the intrauterine hyperglycemic environment during pregnancy, the perinatal period, and the long-term postnatal period on offspring has not yet been fully investigated. The Hyperglycemia and Adverse Pregnancy Outcomes (HAPO) study and the HAPO Follow-up Study (HAPO FUS), which was conducted in 2000, enrolled multicultural and multiracial pregnant women from 15 centers in nine countries to present a “raw” picture of the effects of hyperglycemia during pregnancy on perinatal outcomes. The results of the study showed that the proportion of the GDM group with abnormal glucose metabolism more than 10 years after delivery was more than 50%, which was significantly higher than that of 20% in the control group. The prevalence of impaired glucose tolerance (IGT) in the offspring from mothers with GDM and those without GDM was 10.6% and 5.0%, respectively, and maternal GDM was an independent risk factor for the development of IGT in offspring. In addition, there was a significant correlation between GDM in mothers and their offspring having obesity in childhood [3,4]. Large population-based studies have shown that offspring from GDM mothers are at a significantly higher risk of obesity in childhood [5,6,7]. Lipids associated with de novo adipogenesis are elevated in pregnant women with GDM compared with normoglycemic women (e.g., triglycerides, fatty acid, and specific diglycerides), and this increase is strongly correlated with maternal hyperglycemia and insulin resistance severity. Nine adipogenesis-related species were significantly associated with large for gestational age (LGA) and birth weight percentile, and the correlation between specific diglycerides (DG, 32:0) and triglycerides (TG, 46:0), (46:1) and neonatal abdominal circumference persisted after adjustment for maternal fasting glucose [8]. The glucose level during pregnancy is an important index to predict the future metabolic status of mothers and their offspring, and how to reverse the effect of GDM on the metabolism of the mother and child deserves more extensive study.

The gut microbiota colonizes the host gut and is known as “the second genome” [9]. The human gut microbiota includes approximately 500~1000 bacterial species. The number of coding genes in the gut microbiota is approximately 2,000,000, which is 100 times as high as that in the host genome [10]. The gut microbiota has a great influence on host health [11]. A growing body of evidence suggests that host gut microbes are involved in the development of multiple metabolic diseases. Type 2 diabetes mellitus (T2DM) is a metabolic disease characterized by pancreatic β-cell dysfunction and peripheral insulin resistance, and several studies have demonstrated a dysregulated gut microbiota in patients with T2DM, as evidenced by an increased abundance of pathogenic bacteria, such as *Clostridium hathewayi* and *Escherichia coli*. Interestingly, the characterization of the patients’ gut microbiota changed throughout the course of diabetes. When the patients were in the pre-diabetic stage, the abundance of the *Clostridium* species was reduced in the intervention group compared to that in the control group. This suggests that there may be complex interactions between the host and gut microbiota in the regulation of glucose metabolism [12]. A meta-study included 23 studies on the relationship between GDM and gut microbiota, 17 of which showed a significant reduction in the alpha and beta diversity of the gut microbiota in pregnant women with GDM [13]. In addition, intervention studies reported an improvement in glucose metabolism in the GDM group after probiotic supplementation, and interestingly, probiotic supplementation also seemed to reduce the risk of GDM in the women [14]. Although the causal relationship between the gut microbiota and disorders of glucose metabolism during pregnancy is still unknown, it is undeniable that the gut microbiota plays an important role in both the prevention of GDM and the improvement of adverse pregnancy outcomes associated with maternal hyperglycemia. Here, we focused on the mechanisms by which GDM affects offspring metabolism via the gut microbiota as well as early-life interventions targeting gut microbiota improvement.

## 2. Maternal Metabolism, Gut Microbiota, and Offspring Metabolism

### 2.1. Gut Microbiota Links between Maternal and Offspring Metabolism

#### 2.1.1. Establishment of the Fetal Gut Microbiota and Influencing Factors

Previously, the common perception was that gut microbes in newborns were vertically transmitted by their mothers during labor and delivery. However, as research has progressed, the notion that the uterus is a sterile environment has been shattered, and microbiota are present in the endometrium. Although there is a limited understanding of fetal bacterial profiles in relation to intrauterine profiles, the discovery of the concept of intrauterine profiles offers more possibilities to explain the establishment of fetal gut microbiota. Interestingly, the oral and gut microbiota have also been found to be associated with fetal health [15]. Postnatal feeding methods are equally important factors influencing the gut microbiota of infants. Breastfed infants are characterized by a bacterial profile dominated by bifidobacterial colonization. Compared with breastfeeding, formula-fed infants show variability in gut microbiota, which may be due to the prebiotic effect of oligosaccharides in breast milk [16,17]. Oligosaccharides in breast milk aid in the growth of beneficial bacteria [18], while acetate and lactate produced by *Bifidobacteria* limit the growth of pathogenic bacteria such as *Escherichia coli* [19]. In addition, breast milk contains bacteria such as *Staphylococcus*, *Streptococcus*, *Bifidobacterium*, and *Lactobacillus* that are continuous sources for the development of the gut microbiota in infants [20]. Thus, the establishment of the infant gut microbiota involves a combination of factors, including the microbiota of the placenta, umbilical cord blood, amniotic fluid, and fetal meconium, all of which may be involved in the development of the neonatal gut microbiota [21,22,23]. Unlike the relatively stable microbial environment of adults, the gut microbiota in infants and toddlers is unstable, and it is not until the age of three years that a stable gut microbiota similar to that of adults is established in children. Therefore, given the low diversity and high individual variability of the gut microbiota in infants and young children, the early developmental environment is highly susceptible to influencing their development. Maternal lifestyle factors, prenatal exposures, the mode of delivery, and feeding practices may all influence the health of offspring by altering the gut microbiota [17,24]. Differences in the gut microbiota of offspring born to obese and lean mothers were found and were still observed at the age of one year [25,26]. The fecal *Bacteroides* and *Staphylococcus* levels in six-month-old infants were higher in those from overweight mothers [25]. Even in 18- to 27-month-old toddlers, the fecal *Faecalibacterium* spp., *Eubacterium* spp., *Oscillibacter* spp., and *Blautia* spp. levels were different between those with obese and non-obese mothers [26].

#### 2.1.2. Links between Maternal Gut Microbiota and Offspring Health

Under normal physiological conditions, the gut microbial ecosystem regulates the immune system and metabolism, thereby contributing to nutritional homeostasis. An imbalanced gut microbiota can lead to the development of a variety of adverse metabolic diseases, including obesity, diabetes, and cardiovascular disease [27]. Substances are exchanged between the mother and fetus through the placenta and umbilical cord blood to provide nutritional support for early fetal growth and development. Therefore, any change in a mother’s metabolic status may affect the early developmental environment of her fetus through substance exchange, thereby affecting the health of her child later in life. Maternal dietary changes during pregnancy can affect fetal growth and development through the gut microbiota, and some specific maternal gut bacterial species are necessary for the maintenance of the immune system and neurodevelopment in fetuses [15]. A Chinese cohort study found that infants born from GDM mothers had reduced β-diversity of the meconium microbiota and increased BMI Z scores at one year of age [28]. To investigate the role of gut flora in the influence of GDM on fetal BMI, the researchers performed regression analyses on the gut flora of GDM and normoglycemic mothers. The results showed a coabundance group depleted in the GDM group was negatively correlated with 12-month infant BMI. Therefore, gut flora may be an important mediator in the influence of GDM mothers on fetal development.

#### 2.1.3. Relationship between GDM and Dynamic Changes in Offspring Gut Microbiota

It is well established that maternal hyperglycemia affects the diversity of gut microbiota in pregnant women, while questions remain about the effect of GDM on the composition of infant gut microbiota. In a prospective cohort study that included 73 pregnant women, of whom 34 had GDM and 39 did not, the microbial composition of feces from their infants at one month of age (M1) and six months of age (M6) was analyzed to investigate the relationship between GDM and the dynamics of the infants’ gut microbiota. The results showed that there were no significant differences in diversity and composition at M1 between the GDM and non-GDM groups. However, there were 11 species at M6 in the infants’ gut microbiota in the GDM group, with lower levels of diversity. More importantly, the dynamics of α-diversity from M1 to M6 differed significantly depending on GDM status [29]. This suggests that the dynamic development of the gut microbiota after birth remains closely linked to maternal metabolic status. As an indispensable factor in maintaining metabolic status, the establishment and maintenance of gut microbiota homeostasis may affect the lifelong health of offspring. GDM, a prevalent and common disease, threatens metabolic health in both mothers and their offspring. This issue has broad research prospects on the mechanisms by which GDM affects the gut microbiota of offspring and related early interventions.

### 2.2. The Mechanism by Which Maternal Hyperglycemia Affects the Gut Microbiota and Programs Metabolism in Offspring

Maternal hyperglycemia can affect the stabilization of the gut microbiota; in turn, the microbiota is equally involved in the development of metabolic disorders in offspring, although the interrelationship between gut microbiota and GDM is currently unknown. In this section, we focus on the updated mechanisms by which GDM affects offspring metabolism through the gut microbiota.

#### 2.2.1. Effects of Early-Life Gut Microbial Changes on Metabolism Later in Life

Initial gut microbiota colonization in infants has a critical impact on later metabolic health; in 2012, Professor Cohen at the University of Rhode Island proposed the “Restaurant” hypothesis that intrauterine malnutrition alters metabolic signaling pathways by shaping the initial gut microbiota of infants, particularly *Escherichia coli* [25,26]. Longitudinal studies have shown that a high abundance of *Lactobacillus* spp. and a low abundance of *Mycobacterium* spp. in the gut of infants from 0 to 3 months of age predicts the risk of obesity and overweight in childhood [30] and that characteristics of the composition of the gut microbiota in early infancy correlate with body weight at 7 years of age [31]. In addition, both cohort and animal studies have shown that feeding a high-fat diet (HFD) to offspring leads to a reduction in the diversity of their gut microbiota, with a decrease in the abundance of *Campylobacter jejuni* and *H. pylori* and an increase in the abundance of *Trichospirillaceae* and *Clostridia* spp. Even when the offspring were weaned and returned to a normal diet, this change could not be reversed [32,33,34].

#### 2.2.2. Maternal Hyperglycemia Programs Offspring Metabolism via Metabolites

##### The Role of Metabolites in Offspring Glucose Metabolism

Currently, metagenomics and 16S ribosomal RNA (rRNA) sequence analyses can provide advanced technical support to study gut microbes on a large scale [27]. However, our understanding on the physiological functions of the gut microbial system is still very limited. In recent years, to better study the complex interactions between the gut microbiota and the development of diseases, many new analysis techniques have been developed to provide more important information for the study of gut microbiota, including metabolomics. Metabolomics is defined as the dynamic, multiparameter metabolic response of an organism that is perturbed by genetic or environmental alterations. With its powerful analytical capabilities, metabolomics has been widely used in the screening of biomarkers for diseases and in the diagnosis and prognosis of diseases [27].

- The role of circulating metabolites in offspring glucose metabolism

A prospective study in China analyzed the changes in the fecal microbiota and plasma metabolome of pregnant women with GDM in mid-pregnancy. The results of this study showed that changes in the maternal fecal microbiota were associated with changes in plasma glycerol, lactate, proline, and methylmalonic acid levels in women with GDM [35]. In the GDM Mother and Child cohort study, meconium microbiota and metabolites were examined. The neonates in the GDM group not only had a decreased diversity of gut microbiota but also several metabolite changes in their meconium, showing a similar trend of alterations to maternal serum metabolites [36]. Gut microbes can break down and metabolize nutrients in the gut, producing substances such as vitamins, amino acids, and short-chain fatty acids (SCFAs). These metabolites can diffuse through the gut into the circulation and affect the metabolism of other organs, including the liver, kidney, and brain. The effects of circulating metabolites make it difficult to accurately determine the metabolic pathways [37]. Studies on germ-free (GF) mice have found that the serum levels of bacterial-derived metabolites such as SCFAs, macronutrients, and choline are influenced by the gut microbiota [38,39].

- The role of gut microbiota metabolites in glucose metabolism in offspring

The gut microbiota metabolome is an important tool for determining the pleiotropic effects of the gut microbiota on the host. Metabolites can influence different signaling pathways, including through circulation, and thus participate in several physiological responses in the host. An increasing number of studies suggest that specific metabolites, including branched-chain amino acids (BCAAs), in the gut microbiota are strongly associated with the risk of insulin resistance [40,41,42]. A study from the University of Washington found that maternal weight gain during pregnancy, a risk factor for long-term metabolic diseases in offspring, was associated with altered fecal carbohydrate degradation and vitamin synthesis pathways in offspring [43]. Animal studies have shown that maternal glucose metabolism phenotypes and the gut microbiome can be “transmitted” to offspring [44]. In this study, murine offspring from hyperglycemic dams showed insulin resistance and impaired glucose tolerance. The fecal microbiota structure of the offspring was similar to that of their mothers. Further analysis showed reduced gut *Bifidobacteria* abundance and fecal SCFA levels in the offspring of dams with GDM. To investigate the effect of gut microbiota vertical transmission on offspring metabolism, researchers prevented vertical transmission by performing cesarean section (CS). Impaired pancreatic β-cell secretory function and insulin resistance were ameliorated in the offspring from dams with GDM in the CS group. The levels of fecal metabolites were also affected by CS and were strongly correlated with glucose metabolic profiles. Moreover, the influence of environmental factors after birth may also play an important role in the development of the gut microbiota. To explore this effect, the study cross-fed pup mice, which similarly ameliorated the negative effects of GDM on insulin sensitivity and islet function in the offspring. Interestingly, cross-feeding had a greater effect on the vertical transmission of the gut microbiota than cesarean delivery. This phenomenon may generate new perspectives, i.e., postnatal care may play a more important role in the establishment of the neonatal gut microbiota.

#### 2.2.3. Epigenetic Links between GDM and the Gut Microbiome and Their Effects on Offspring Glucose Metabolism

In 1942, the concept of “epigenetics” was first introduced to study the interactions between genes and their transcripts. Currently, epigenetics is defined as the molecular modifiers and processes surrounding DNA that regulate genomic activity while remaining stable in mitosis and independent of the DNA sequence. DNA methylation, histone modifications, and noncoding RNAs are the three classical processes that are considered “epigenetic”. Growing evidence shows that epigenetics may explain early life nutrition and the development of glucose metabolism disorders in offspring in later life, including hypertension, dyslipidemia, and T2DM [45]. In recent years, the relationship between the gut microbiota and epigenetic modification has become a research hotspot. Specific patterns of change in epigenetic modifications affected by exposure to certain gut bacterial species may be a potential mechanism for the complex interactions between the gut microbiota and the epigenome. A pilot study explored the dominant microbiota as a factor in epigenetic modification. Based on their dominant gut bacterial phyla, pregnant women were categorized into the high *Bacteroidetes* phylum (HighBact) group and the high *Firmicutes* phylum (HighFirm) group. A blood DNA methylation pattern analysis revealed that mothers in the HighBact group had a total of 568 genes with higher promoter methylation and 245 genes with lower promoter methylation than those in the HighFirm group [46]. A similar phenomenon was found through an umbilical cord blood analysis. Changes in the methylation of the diabetes susceptibility genes *UBE2E2* and *KCNQ1* in umbilical cord blood were correlated with the proportion of maternal gut *Firmicutes* [47]. Population studies have revealed that a high gut Bacteroidetes-to-Firmicutes ratio in T2DM patients may be an important trait involved in abnormal glucose metabolism [48]. In addition, some specific bacteria in the *Firmicutes* phylum, such as *Faecalibacterium prausnitzii*, are a major source of butyrate, which can regulate gene expression through histone modification [49]. Thus, the ways in which gut microbiota regulate epigenetic modifications are diverse and complex. In a prospective, randomized study of maternal and infant nutrition and probiotics, probiotic supplementation was found to significantly reduce maternal blood DNA methylation levels at 37 gene promoters and increase DNA methylation levels at one gene promoter. The offspring in the probiotic group had 68 gene promoters that were significantly affected and had lower levels of DNA methylation. The probiotic-supplemented mothers and their children had lower promoter methylation and increased transcriptional activity of insulin-like growth factor-binding protein 1 (IGFBP1). IGFBP1 concentrations are associated with diabetes, and increasing IGFBP1 concentrations may be a beneficial effect of probiotic supplementation on glucose metabolism [50].

#### 2.2.4. The Role of Circadian Rhythm–Gut Microbiota Interactions in Offspring Glucose Metabolism

Among the lifestyle risk factors that may contribute to glucose metabolism disorders, circadian disorders are receiving increasing attention in scientific research. Circadian rhythms are driven by a molecular biological clock that is present in almost every cell in the human body. By controlling the secretion of melatonin, circadian rhythms regulate sleep cycles [51]. The link between circadian rhythm disruption and metabolism is well established. With the disruption of central and peripheral circadian rhythms, animal models have developed features of metabolic syndrome, such as hyperglycemia, adipocyte hypertrophy, and obesity [52,53,54,55]. The results of these metabolic disorders in rodents are also highly generalized in humans with disrupted circadian rhythms. Humans with variants and polymorphisms of cryptochrome 2 (CRY2) and period circadian regulator 2 (PER2), which are key circadian rhythm genes, exhibit hyperglycemia [56,57]. Maternal peripheral circadian rhythm-related gene expression disorders during pregnancy lead to downstream changes in the circadian expression of specific metabolic genes [58]. Thus, when circadian rhythms are disrupted, pregnant women are at a high risk of metabolic disease. In addition, intergenerational studies have found that the fetal abdominal circumference z score of offspring is associated with maternal circadian regulation disruption in women with GDM [59]. Recent studies have found that the gut microbiome also exhibits circadian variation and that there are dynamic interactions between the host circadian system and gut microbiota. Circadian rhythm disruption alters the gut microbiota structure, thereby increasing susceptibility to metabolic diseases [51].

#### 2.2.5. The Role of Gut Microbiota in Intergenerational Glucose Metabolism Induced by Inflammation

Insulin resistance is a major manifestation of all types of diabetes. There is growing evidence that an abnormal inflammatory state in the body is closely associated with insulin resistance. A low-grade inflammatory response leads to the proliferation of cytokines such as tumor necrosis factor alpha (TNF-α), interleukin 6 (IL-6), and lipocalin. These cytokines can cause systemic insulin resistance by activating signaling pathways such as protein kinase C (PKC) and mTOR/S6K [60]. The mechanism by which the low-grade inflammatory state arises is currently unknown, but recent studies suggest that gut microbiota may be an important link involved in the development of an abnormal inflammatory state. Diet-induced changes in gut microbiota can increase the absorption and recycling of lipopolysaccharides and BCAAs and decrease SCFAs. A decrease in SCFAs can increase intestinal permeability, whereas LPS can induce severe inflammatory responses [61]. This may be one of the mechanisms by which intestinal microbiota are involved in inducing inflammatory responses. In addition, a low-grade inflammatory state has been suggested to have an important influence on the formation as well as the development of GDM. A recent study suggests that GDM may be driven by gut-microbiota-induced inflammation, with women with GDM exhibiting altered gut microbiota and elevated levels of inflammatory cytokines in early pregnancy [62]. After transplantation of GDM as well as control fecal samples into GF female mice, the differences in microbial communities between GDM fecal receptor and non-GDM fecal receptor mice were consistent with those observed among pregnant women, and in addition, the GDM fecal receptor mice exhibited an impaired glucose tolerance phenotype [62]. Interestingly, IL-6 (consistent with findings in women with GDM) was elevated in GDM the fecal receptor mice, along with interleukin 10 (IL-10). IL-6 may be an important mediator in the pathogenesis of GDM induced by inflammation of the gut microbiota [62]. A high fructose intake during pregnancy promotes the development of insulin resistance (IR) in pregnant and postnatal mice and their offspring through the activation of the NF-κB-NLRP3 inflammasome pathway. IL-6, interleukin 17 (IL-17), interleukin 1β (IL-1β), and interleukin 18 (IL-18) levels were significantly elevated in the offspring mice [63]. Another study exploring the interventional effects of proanthocyanidins (PA) on IR in GDM mice found that PAs reduced the expression of IL-6, TNF-α, IL-17, and the c-reactive protein (CRP) and ameliorated IR [64]. However, the effects of PA on the amelioration of IR were significantly attenuated, and the inhibition of inflammation was blocked in intestinal-microbiota-deficient (IFD) mice. The offspring mice with GDM in terms of the area under curve (AUC) of the oral glucose tolerance test (OGTT), the AUC of the insulin tolerance test (ITT), and serum IL-17 and CRP concentrations were significantly elevated [64]. Although no role of PA intervention in offspring glucose metabolism was observed [64], which may be related to the amount of PA passing through the placenta as well as the timing of the study observations, it is undeniable that inflammation may have an important role in intergenerational glucose metabolism and that the gut microbiota may be a target for intervention in inflammation-induced metabolic disorders.

## 3. Effects of Early-Life Interventions Targeting Gut Microbiota on Glucose Metabolism in Offspring

### 3.1. Effect of GDM Therapy on the Gut Microbiota and Glucose Metabolism in Offspring

#### 3.1.1. Insulin

Gestational hyperglycemia may not only contribute to adverse birth outcomes, including perinatal and neonatal complications, but also affect the metabolic health of offspring in adulthood [65]. Therefore, the timely diagnosis of GDM and achieving optimal glycemic control during pregnancy in the shortest time possible are imperative for blocking adverse intergenerational glucose metabolic effects. Currently, the first-line treatment for GDM includes a combination of nutritional therapy and exercise [66]. When lifestyle interventions fail to control blood glucose levels, pharmacological therapy is introduced. Insulin therapy is the first-line medication therapy to control hyperglycemia during pregnancy [66]. Insulin does not cross the placenta, but it can bind to the insulin receptor, a specific receptor in trophoblast membranes, thereby activating the insulin signaling pathway. Therefore, the levels of activated insulin mediators in the placentas of women with GDM may influence fetal metabolism [67,68]. A controlled study in Spain investigated the effects of insulin on placental lipid carriers and insulin mediators in women with GDM controlled by diet or insulin. The results showed that exogenous insulin therapy could promote the phosphorylation of placental insulin mediators by activating the insulin cascade in women with GDM receiving insulin treatment, thereby elevating specific fatty acid carriers and ultimately contributing to increased fatty acid levels in the placenta [69]. Therefore, the use of insulin for glycemic control may require concomitant attention to maternal blood lipid levels to avoid the excessive transfer of fat to the fetus through the placenta [69]. To investigate whether insulin treatment is beneficial to the long-term metabolism of offspring in adulthood, Hong Zhu et al. constructed a diabetic mouse model and treated the mice with insulin [70]. The results of the study showed that insulin treatment had a significant protective effect on offspring exposed to maternal hyperglycemia caused by glucose intolerance and obesity. However, when the offspring mice were fed a HFD, insulin treatment failed to protect their glucose metabolism. The effect was sex-specific, with male mice exhibiting more severe glucose metabolism disorders. To investigate why this phenomenon occurs, experiments were performed to analyze the genome-wide DNA methylation of pancreatic islets in the offspring. Male offspring were found to have hypermethylated regions in genes associated with impaired insulin secretion. This finding suggests that GDM induces glucose intolerance in offspring through a methylation-mediated epigenetic mechanism and that insulin treatment for GDM does not restore altered DNA methylation in offspring pancreatic islets. The gut microbiota also plays a role in the effect of insulin therapy on glucose metabolism in offspring. In a population study, pregnant women were divided into a healthy control group (non-GDM group), a dietary treatment control group (GDM-D group), and an insulin treatment group with dietary control failure (GDM-I group) to compare the effects of dietary control as well as insulin treatment on the gut microbiota of pregnant women with GDM and their newborns [71]. Alterations in the gut microbiota were detected before treatment. The results showed higher proportions of *Clostridiales*, *Lactobacillus*, and *Bacteroidetes* in the GDM-I group than in the non-GDM and GDM-D groups. However, these alterations were ameliorated by insulin treatment in the second trimester, and the levels of these three bacteria were significantly reduced in the GDM-I group. *Enterobacteriaceae* bacteria in the feces of neonates from the GDM-I group were significantly reduced. Interestingly, neonates born to mothers in the GDM-D group had an increased F/B ratio, which was consistent with their mothers’ predelivery situation.

#### 3.1.2. Metformin

Metformin (MT) is an antidiabetic agent widely used in patients with T2DM and obesity. Although MT has been widely used to treat GDM, the effect of maternal MT treatment on the long-term development of offspring remains unclear. During pregnancy, MT increases brain weight as well as total thoracic and abdominal visceral weight in intrauterine growth restriction (IUGR)-affected, small-for-gestational-age (SGA) offspring [72]. Maternal MT treatment during pregnancy and lactation ameliorates the negative effects of a maternal HFD on offspring, and the underlying mechanism may be that MT improves skeletal muscle development through the AMPK/mTOR pathway [73]. In addition, there are sex differences in MT treatment, with females having a smaller body size and males having lower relative weights of major internal organs [74]. Thus, MT has some beneficial effects on fetal growth and development. In terms of glucose metabolism in offspring, metformin treatment during pregnancy leads to an increase in pancreatic β-cell mass in offspring at birth and an increase in insulin secretion in offspring during adulthood [75]. In addition, MT treatment during lactation resulted in offspring weight loss and improved glucose tolerance in male offspring [76]. A study in China explored the role of the gut microbiota in the effect of MT on offspring metabolic phenotypes [77]. Treatment with MT restored the body fat composition in offspring whose mothers were fed an HFD and reduced the inflammatory response in the gut. *Lactobacillus* spp. abundance was elevated in MT-treated male offspring, whereas female offspring were characterized by elevated levels of *Clostridium* spp. However, it has been shown that maternal MT treatment leads to adipocytosis in male offspring [78] without altering the metabolic effects [79].

### 3.2. Modulation of Offspring Glucose Metabolism by Gut Microbiota Therapy

#### 3.2.1. Prebiotic/Probiotic Supplements

Probiotics are a class of living, beneficial microorganisms that, when properly ingested, help to maintain a stable gut microbial system in the host. Given the important influence of gut microbiota in the pathogenesis of GDM, probiotics could be an effective intervention for the prevention of carbohydrate disorders and GDM [14]. However, the results of previous studies on probiotic interventions and GDM remain controversial. Some clinical trials have shown that probiotic supplementation is beneficial in improving glucose metabolism and insulin sensitivity and reducing the risk of elevated glucose levels in pregnant women, with a lower rate of excessive weight gain [80,81]. Supplements containing Lactobacillus acidophilus in GDM mothers may also reduce birth weight in newborns [82]. Animal studies have also shown that probiotic (Lactobacillus acidophilus, Bifidobacterium longum, and Enterococcus faecalis (BiLaEn)) interventions improved glucose metabolism, the gut microbiota, and gut permeability in pregnant rats, and offspring pancreatic islet β-cell differentiation and development were also improved [83]. In addition, prebiotics, as compounds that modulate the composition and activity of the gut microbiota, were also found to improve glucose metabolism through the offspring liver lncRNA Serpina4–ps1/let-7b-5p/Ppargc1a axis [84]. However, other clinical trials have found that probiotics not only fail to improve adverse pregnancy outcomes but also increase the risk of complications such as gestational hypertension [14,80]. Although the results of some studies seem to be very supportive of the efficacy of probiotics, there are also studies that have reported opposite results.

#### 3.2.2. Fecal Microbiota Transplantation (FMT)

Fecal microbiota transplantation (FMT) occurred in China as early as the fourth century, when people used fecal-based medicinal juice to treat patients with severe diarrhea. The use of fresh or fermented fecal suspensions to treat patients with gastrointestinal disorders such as diarrhea, constipation, and abdominal pain was not documented until the Ming Dynasty in China in the 16th century [85]. In 1958, *Pseudomembranous colitis* was also cured by FMT, which was the first reported documented treatment of disease by FMT [86]. Since then, studies on the therapeutic utility of FMT have been conducted, and an increasing number of follow-up case studies have demonstrated the effective role of FMT in the treatment of diseases such as *Clostridioides* difficile infection and refractory ulcerative colitis [87,88]. The application of FMT is no longer limited to the treatment of infectious diseases. In addition, the new direction of linking intestinal microbiota to extraintestinal diseases has further broadened the therapeutic spectrum of FMT. Studies on FMT for the treatment of metabolic diseases date back as far as 2012, when the infusion of intestinal microbiota from lean men into men with metabolic syndrome resulted in increased insulin sensitivity in the recipients [89]. In a transgenerational model, the body weight of sows that underwent transplantation with fecal extracts from high-feed-efficiency pigs was reduced, and offspring had a higher feed efficiency as well as gut bacterial diversity, a higher relative abundance of *Lentisphaera* and *Synergistetes* [90], and decreased carcass weight [91]. In arsenic-exposed sows, the gut microbiota in offspring was affected by FMT and was similar to that of the normal group [92]. In recent years, a growing body of research has affirmed the beneficial effects of FMT in improving obesity and insulin resistance [93]. However, studies to improve glucose metabolism in offspring through FMT are not currently available.

### 3.3. Maternal Dietary Regulation of Offspring Glucose Metabolism

#### 3.3.1. Micronutrient Supplementation

Minerals, trace elements, and vitamins are collectively known as micronutrients. Although small amounts of micronutrients are required by the body, they are essential for normal physiological activities and the maintenance of homeostasis [94]. In 2004, an animal study explored the effects of maternal micronutrient deficiency or surplus on glucose metabolism in offspring. After limiting the maternal intake of multiple minerals and vitamins, the offspring showed early growth retardation, higher body fat, and insulin resistance [95]. Since then, animal and clinical studies on the effects of single micronutrients on the metabolic state of offspring have been conducted. Now, micronutrient deficiency has become an important influencing factor threatening the health of mothers and children. Micronutrient supplementation during pregnancy is expected to be an effective clinical strategy to ameliorate adverse pregnancy outcomes. A meta-analysis showed that high blood 25(OH)D levels were linked with a low risk of GDM [96]. Vitamin D supplementation in women diagnosed with GDM significantly reduced serum fasting glucose, insulin, and homeostasis model assessment of insulin resistance (HOMA-IR) concentrations [97]. The possible mechanisms by which vitamin D supplementation influences the risk of GDM are currently unknown, but its beneficial effects on GDM deserve further investigation. An animal experiment analyzed the effects of different supplementation doses as well as different combinations of micronutrients on offspring metabolism. Pregnant rats were divided into a recommended vitamin (RV) group, a high multivitamin (HV) group, a high folate with recommended choline (HFol) group, and a high folate without choline (HFol-C) group [98]. The response to glucose loading in male offspring from dams in the HV and HFol-C groups was 31% higher than that in the RV group. In female offspring, the response to glucose loading was 29% higher in the HV and HFol-C groups than in the RV group, and the response to glucose loading was 24% lower in the HFol group than in the HV and HFol-C groups. For the composition of gut microbiota, *Shigella*, *Clostridiales*, and *Clostridiaceae* were higher in progeny from dams in the HV group, and *Odoribacter*, *Akkermansia muciniphila*, and *Blautia* were higher in offspring from dams in the HFol and HFol-C group. Thus, unbalanced micronutrients affect offspring metabolism and alter the gut microbiota. The regulation of glucose metabolism in offspring by micronutrients is affected in many ways.

#### 3.3.2. Active Substance Supplementation

##### SCFAs

SCFAs are microbial products produced by intestinal microbiota during the digestion of dietary fiber, including acetic, propionic, and butyric acids. SCFAs are not only involved in the maintenance of intestinal barriers but also act as signaling molecules involved in a variety of metabolic pathways [99]. In terms of intervening in impaired glucose metabolism, butyric acid oral administration increased insulin sensitivity in a mouse model [100]. In addition, in a transgenerational model, SCFA intervention ameliorated hypertension in the offspring of dams fed a high-fructose diet [101]. Acetate is one of the three most common short-chain fatty acids in the body [102]. After comparison with controls, magnesium acetate treatment reduced the birth weight and kidney weight in the male offspring and reduced systolic and diastolic blood pressure and mean arterial pressure in the underfed dams. Moreover, sodium butyrate treatment reduced blood pressure in the offspring of dams fed a tryptophan-free diet and increased the abundance of *Verrucomicrobia* [103]. The effective role of SCFAs in protecting offspring from hypertension could provide us with a theoretical basis for interventions for glucose metabolism in offspring.

##### Genistein

Genistein is a phytoactive substance that has been shown to have beneficial effects in individuals with cardiovascular disease, cancer, bone mineral metabolism disorders, and neurological disorders [104]. As one of the most important components of soy isoflavones, genistein may likewise improve impaired glucose metabolism [105,106]. This may be related to the ability of genistein flavonoids to enhance beta cell proliferation, promote insulin secretion, and regulate the gut microbiota [105]. Genistein was administered to mice three weeks prior to mating, during gestation, and during lactation, during which time glucose metabolism was evaluated, and at the end of the experiment, cecum contents, adipose tissue, and blood samples were taken from the male mice [104]. The offspring glucose metabolism was significantly improved by maternal genistein intervention, with an increase in the abundance of the *genus Rikenella* and a decrease in the abundance of the phylum *Tenebacterium*. Although this experiment only verified the beneficial effects of genistein on glucose metabolism in male mouse offspring, it confirmed the protective role of gut microbiota in genistein metabolism in adulthood.

##### Methyl Donors

Methyl donors are a group of substances that provide methyl groups for biochemical reactions in the human body. Common methyl donors include folic acid, choline, betaine, vitamin D, and methionine. Methyl donors are involved in single-carbon metabolism, an important metabolic pathway in the body that is involved in a variety of biochemical reactions and influences the development of many diseases. Studies have shown that the nutritional intake of methyl donors is strongly associated with a variety of diseases, including T2DM [107]. In a piglet model, maternal methyl donor supplementation (folic acid, methionine, choline, vitamin B6, and vitamin B12) could modify offspring gut microbiota, such as by increasing *Firmicutes* abundance and reducing *Bacteroides* abundance. In addition, the fecal concentrations of acetate, butyrate, and total SCFAs increased in the offspring from the methyl donor supplementation group at twenty-one days of age [108]. However, the concentration of methyl donor supplements and interactions with other supplements were also strongly associated with the effectiveness of the intervention. Maternal folate and choline overload or imbalances lead to an increased risk of obesity in the offspring, and this alteration is associated with a disturbance in the gut microbiota [98].

##### Resveratrol

Resveratrol (RES) is a naturally occurring plant-active substance found mainly in grains, vegetables, legumes, and fruits. RES has diverse physiological and pharmacological properties and has beneficial antiobesity, antidiabetic, anti-inflammatory, and antioxidant properties. Population and animal studies have shown that RES lowers blood glucose and improves insulin sensitivity [109]. RES has a selective modulatory effect on the intestinal microbiota, increasing *Lactobacillus* and *Bifidobacterium* and decreasing *E. coli* and *Enterobacteria* [110]. HFD reduces the *Firmicutes*-to-*Proteobacteria* ratio and *Lactobacillus* spp. abundance in progeny, and RES supplementation restores these changes and prevents HFD-induced hypertension [111].

#### 3.3.3. Mediterranean Diet

The Mediterranean Diet (MD) is a dietary pattern that is low in saturated fats and high in vegetable oils. Currently, based on research and the advancement of guidelines, the MD has been updated to include a high intake of olive oil, vegetables, fruits, grains, nuts, and legumes as well as a moderate intake of fish, other meats, and dairy products [112]. In recent years, a large number of studies have shown the benefits of the Mediterranean diet during pregnancy in improving maternal and infant outcomes. Pregnant women who are more adherent to the MD reduce the risk of developing obesity and cardiovascular diseases in children [113]. When mothers are less adherent to the MD, the fetus may experience growth restriction as well as neonatal insulin resistance [114,115]. MD-based nutrition during pregnancy can help reduce the incidence of gestational diabetes, and pregnant women with GDM are less likely to require insulin therapy and have lower gestational weight gain [116]. In children, following an MD during pregnancy is protective against asthma, allergies, and weight gain [117,118]. Although, for the time being, there are no studies to elucidate the role of gut flora in an MD affecting maternal and infant outcomes, an MD during pregnancy may be a very appropriate intervention to improve the health of the offspring.

### 3.4. Effect of Maternal Lifestyle Regulation on Offspring Glucose Metabolism

#### 3.4.1. Physical Activities

Moderate physical activity during pregnancy is effective in improving glycemic control in some pregnant women. However, physical activity during pregnancy alone may not be sufficient to reduce the risk of GDM [119,120]. A Swedish study showed that healthy pregnant women who exercised during the year before pregnancy had a reduced risk of GDM compared to those who exercised only during pregnancy [121]. In addition, differences in physical activity intensity, maternal health, and dietary status also influence the effects of physical activity interventions. Dayana Mahizir et al. explored whether exercise could ameliorate metabolic and gut microbiota dysfunction exacerbated by a HFD [122]. The results showed that female rats fed an HFD had adverse metabolic responses and an increase in the ratio of Firmicutes to Bacteroidetes in feces. Interestingly, only endurance exercise before and during pregnancy prevented these changes in the gut microbiota. No gut microbiota restoration was observed in pregnant female mice that exercised only during pregnancy. This phenomenon emphasizes the importance of preconception exercise to ameliorate adverse pregnancy outcomes. Therefore, how to plan preconception as well as pregnancy exercise to improve impaired glucose metabolism is an important topic for clinical intervention.

#### 3.4.2. Sleep

Sleep alteration can regulate glucose metabolism and increase the risk of diabetes. Current research suggests that this regulatory mechanism may be related to oxidative stress, inflammation, and cortisol levels [123]. In the first trimester, pregnant women with poor sleep quality have an increased risk of developing GDM [124]. Another meta-analysis suggested that excessive sleep duration in early and mid-pregnancy is also strongly associated with GDM [125]. In addition, sleep disorders are also a risk factor for GDM, and large population studies have shown that women with sleep disorders are at higher risk for GDM than controls [126]. Recent studies have shown that sleep deprivation (SD) can lead to dysregulation of microbial ecology, resulting in neuroinflammation [127]. Pregnant rats were subjected to SD on Days 15–21 of gestation. The gut contents and brain tissue of their offspring were collected at 1, 7, 14 and 56 postnatal days. The offspring intestinal microbiota showed alterations characterized by an elevated abundance of *Firmicutes*. However, elevated IL-1β and TNF-α levels were detected in the brain tissues of the offspring at 56 days of age. A correlation analysis showed that IL-1β and TNF-α levels were positively correlated with the abundance of *Ruminococcus_1* and *Ruminococcaceae_UCG-005*. Sleep disruption, a short or long sleep duration, and sleep disorders in pregnant women have become critical public health issues threatening the health of both mothers and children.

## 4. Conclusions and Future Prospects

**Figure 1:**
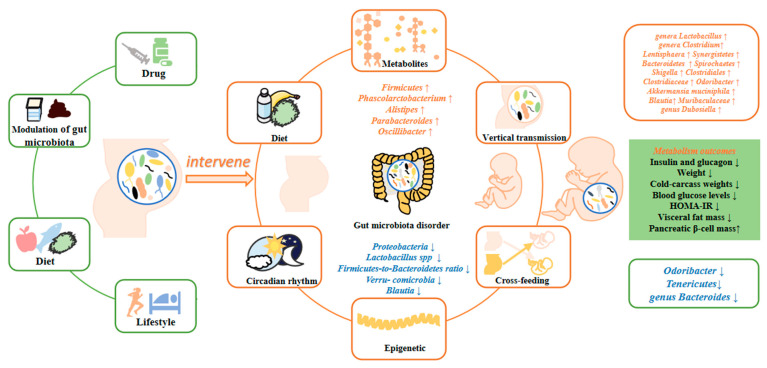
The possible mechanisms and interventions in the role of gut microbiota in gestational diabetes mellitus affecting intergenerational glucose metabolism. (↑, the abundance or metabolic index being upregulated; ↓, the abundance or metabolic index being downregulated).

Hyperglycemia during pregnancy has been shown to affect offspring metabolic health. There is growing evidence that the gut flora plays a role in impaired glucose metabolism. Additionally, altered gut flora in pregnant women with GDM may be a risk factor for glucose metabolism abnormalities in their offspring. In this review, we summarized the possible mechanism of action by which the gut microbiota influences glucose metabolism in offspring (Figure 1 and Table 1). The establishment of the fetal gut microbiota is dependent on vertical transmission from mothers, and when the maternal gut microbiota shifts, the offspring gut microbiota may follow the same trend of alteration. In addition, metabolites in offspring, which are products of the gut microbiota, are also influenced by maternal gut microbiota alteration trends. Early epigenetic modifications are key determinants of metabolic health in later life, so specific patterns of changes in epigenetic modifications following exposure to certain bacteria may be a potential mechanism for the complex interactions between the gut microbiota and the epigenome. The negative impact of altered circadian rhythms on human metabolism has been extensively studied in recent years. The microbiome is similarly subjected to circadian rhythm variations, and disruption of host circadian rhythms affects the composition of the microbiota. The gut microbiota may be a possible mechanism by which circadian rhythm disruption is involved in the pathology of adverse pregnancy outcomes. In summary, the effects of the gut microbiota on glucose metabolism in offspring are multifaceted and complex, and more studies are needed to elucidate their mechanisms of action to provide more data for clinical practice.

Currently, clinical glycemic control through diet and medication is common, and clinical studies on the effects of other interventions on offspring metabolism are still needed. We summarized the beneficial effects of pharmacological interventions, microbiota modulation, active substance supplementation, and lifestyle interventions on glucose metabolism in offspring (Figure 1 and Table 2), and although population studies and experimental animal studies are still limited, the potential for improving glucose metabolism in offspring by interfering with the composition of the intestinal microbiota is promising. More detailed population and animal studies are needed to identify feasible intervention strategies to reduce the threat of GDM to the health of future generations.

## Figures and Tables

**Table 1:** The cohort studies on the relationship between GDM gut microbiota and progeny metabolism.

Race	Diet/Treatment	Sample Size	Metabolism Outcomes	Gut Microbiota	Specific Gene Expression	Ref.
**Observational study**						
**Asian**	N/A	GDM (*n* = 20):non-GDM (*n* = 20)	Blood glucose levels ↑ in GDM group	Alpha and beta diversity ↓; *Blautia* ↓, *Phascolarctobacterium, Alistipes, Parabacteroides, Eubacterium coprostanoligenes_group, Oscillibacter, Paraprevotella*, and *Ruminococcaceae* *NK4A214_group* ↑	Methylmalonic acid and glycerol ↓, galactitol, lactic acid, and proline ↑ in the plasma metabolome of GDM.	[35]
**Asian**	N/A	GDM (*n* = 147): non-GDM (*n* = 271)	Blood glucose levels ↑ in GDM group	Alpha diversity ↓ in neonates; relative abundance of *Proteobacteria* ↓ and *Firmicutes* ↑	The abundations of GPC, glycholic acid, rhamnose ↓, riboflavin, and taurine ↑ in meconium and maternal blood in GDM group	[36]
**European**	N/A	GDM: HighFirm group (*n* = 4): HighBact group (*n* = 4)	N/A	HighBact: *Bacteroidetes* and *Proteobacteria* ↑ HighFirm: *Firmicutes* ↑	The promoters of 568 genes methylated ↑ and the promoter of 245 genes methylated ↓ in HighFirm group than in HighBact group	[46]
**Asian**	N/A	non-GDM (*n* = 10)	N/A	*Firmicutes* ↑	The UBE2E2 and KCNQ1 methylation rates in umbilical cord samples were associated with the proportion of Firmicutes in the maternal gut	[47]
**Asian**	N/A	GDM (*n* = 44): non-GDM (*n* = 350)	N/A	The microbial communities significantly different	IL-4, IL-6, IL-8, TNF-α	
**Intervention study**						
**Asian**	Insulin	Non-GDM (*n* = 33): GDM-I (*n* = 8): GDM-D (*n* = 30)	Blood glucose levels↑ in GDM group	Maternal *Clostridiales*, *Lactobacillales*, and *Bacteroidetes* ↓ in the GDM-I; *Enterobacteriaceae* ↓ in the first feces of the GDM-I group	N/A	[71]

Abbreviation: GDM-D, dietary treatment of gestational diabetes mellitus; GDM-I, insulin treatment of gestational diabetes mellitus; GPC, glycerophosphocholine; HighBact, high Bacteroidetes phylum; HighFirm, high Firmicutes phylum; IL-4, interleukin 4; IL-6, interleukin 6; IL-8, interleukin 8; KCNQ1, potassium voltage-gated channel subfamily Q member 1; non-GDM, non-gestational diabetes mellitus; TNF-α, tumor necrosis factor-α; UBE2E2, ubiquitin-conjugating enzyme E2. ↑, the expression or concentration being upregulated; ↓, the expression or concentration being downregulated.

**Table 2:** Animal studies on potential mechanism of VD deficiency during pregnancy on offspring obesity.

Animal Models	Diet/Intervene	Metabolism Outcomes	Gut Microbiota	Specific Gene Expression/Metabolite	Ref.
C57BL/6 mice	HFD	C-section group: serum insulin ↓	C-section group: *Muribaculaceae* and *genus Dubosiella*↑; *genus Bacteroides* ↓	BCAAs, salicylic acid, and isobutyric acid ↑, dipeptides containing proline ↓ in the C-section delivered offspring from mHF	[44]
Sprague-Dawley rats	300 mg/kg/d MT treatment	Body weight in HF-MT dams ↓; RP fat weight in HF-MT offspring ↑	Male offspring: *genera Lactobacillus* ↑ Female offspring: *genera Clostridium* ↑	IL-6, Hmgb1, and TLR2 in HF-MT dams ↓; mRNA expression of Ocln in HF-MT dams ↑	[77]
C57BL/6J mice	BiLaEn-L group (6.4 × 10^6^ CFU/day); BiLaEn-H group (1.28 × 10^7^ CFU/day)	Prevent the expression of insulin and glucagon in offspring islets ↑	In mothers: the amount of *norank_f_Desulfovibrionaceae* and *norank_f_Oscillospiraceae* ↓, the amount of *Lactobacillus* and *Faecalibaculum* ↑	pS6 ↑ after BiLaEn treatment in islets	[83]
Pigs	200 mL FMT	Body weight and cold-carcass weights ↓	*Lentisphaera* and *Synergistetes* ↑	Butyric acid ↓, isobutyric acid↑in fecal	[90]
Pigs	200-mL FMT; 8-mL FMT	FMTP sows: carcass weight ↓	FMTP sows: *Oribacterium, Tenericutes, Candidatus Saccharibacteria*, and *Anaerovibrio* ↑ FMTP offspring: *Bacteroidetes* and *Spirochaetes* ↑; *Tenericutes*, *Chlamydiae*, and *Actinobacteria* ↓	Propionic, butyric acid ↑ and isobutyric acid ↓ in the ileum	[91]
Sprague-Dawley rats	75 mg/L sodium arsenit in water; 1 mL/100 g FMT	Prevent latency ↑	*g_Prevotella, g_UCG_005* ↑, *p_Desulfobacterota, g_Eubacterium_xylanophilum_group* ↓	Expression of LPS, TLR4, Myd88, and NF-κB in colonic and striatal tissues ↓	[92]
Wistar rats	RV; HV:10-fold the recommended multivitamin mix; HFol: 10-fold folic acid with recommended choline; Hfol-C: 10-fold folic acid	Male offspring: the HV, Hfol, and Hfol-C body weight ↑ than the RV All offspring glucose response ↑ to a glucose load in HV compared to Hfol-C than RV	HV offspring: *Shigella, Clostridiales, Clostridiaceae* ↑ and *Odoribacter* ↓; Hfol and Hfol-C offspring: *Odoribacter, Akkermansia muciniphila*, and *Blautia* ↑	Male offspring: butyric acid ↓ in the HFol and HFol-C groups compared to RV Female offspring: acetic acid ↑ in the HV and HFol-C groups	[98]
Sprague–Dawley rats	Sodium butyrate 400 mg/kg/d in water	The KW and the KW-to-BW↓; SBP, diastolic BP, and MAP ↓	Prevent *Verrucomicrbia* ↓	Prevent mRNA expression of AGT and ACE ↑	[103]
C57BL/6 rats	0.6 g/kg diet genistein	Blood glucose levels, serum insulin concentrations, HOMA-IR and visceral fat mass ↓	*Tenericutes* ↓, *Rikenella* ↑	N/A	[104]
Duroc × Erhualian gilts	MET-supplemented diet	Body weight at weaning ↑	*Dialister*, *Megasphaera*, *Turicibacter*, *Akkermansia*, *Weissella*, and *Pediococcus* ↑	Individual and total SCFAs of 21-day piglets ↑ in fecal	[108]
Sprague Dawley rats	Resveratrol (50 mg/L) in water	SBP, diastolic BP, and MAP ↓	Prevent *Firmicutes*-to-*Proteobacteria* ratio and *Lactobacillus* ↓	Prevent renal Nrf2 mRNA expression ↓	[111]
Wistar-Kyoto rats	Exercise	Fetal weight and pancreatic β-cell mass ↑	Prevent the *Firmicutes*-to-*Bacteroidetes* ratio ↓	N/A	[122]
Sprague-Dawley rats	SD	N/A	*Firmicutes* ↑	IL-1β, TNF-α ↑ in the brains	[127]

Abbreviation: ACE, angiotensin-converting enzyme; AGT, angiotensinogen; BCAAs, branched-chain amino acids; BiLaEn-H, high-dose *Lactobacillus acidophilus*, *Bifidobacterium longum*, and *Enterococcus faecalis*; BiLaEn-L, low-dose *Lactobacillus acidophilus*, *Bifidobacterium longum*, and *Enterococcus faecalis*; BW, Body weight; FMTP, FMT procedure; HF-MT, HF-fed dams treated with MT; HFol, high folate with recommended choline; HFol-C, high folate without choline; Hmgb1, high mobility group box-1 protein; HV, high multivitamin; IL-1β, interleukin-1β; IL-6, interleukin 6; KW, kidney weight; LPS, serum lipopolysaccharide; MAP, mean arterial pressure; mHF, mice with HFD feeding; MT, metformin; Myd88, myeloid differentiation factor 88; NF-κB, nuclear transcription factor-κB; Nrf2, nuclear factor erythroid2-related factor 2; Ocln, occludin; PND, postnatal day; pS6, protein S6; RP, retroperitoneal; RV, recommended vitamin; SAT, subcutaneous adipose tissue; SBP, systolic BP; SC, subcutaneous; SCFAs, short-chain fatty acids; SD, sleep deprivation; TLR2, toll-like Receptor; TNF-α, tumor necrosis factor-α; VAT, epididymal visceral adipose tissue. ↑, the expression or concentration being upregulated; ↓, the expression or concentration being downregulated.

## Data Availability

Not applicable.
